# The association between physical symptoms and self-care behaviours in heart failure patients with inadequate self-care behaviours: a cross-sectional study

**DOI:** 10.1186/s12872-023-03247-2

**Published:** 2023-04-22

**Authors:** Xu Liu, Li Liu, Yan Li, Xi Cao

**Affiliations:** 1 Department of Infectious Disease, The Fifth Affiliated Hospital, Sun Yat-Sen University, Zhuhai, Guangdong Province China; 2 Xiangya Nursing School, Central South University, Changsha, Hunan Province China; 3 School of Nursing, The Hong Kong Polytechnic University, Hong Kong S.A.R, China; 4 School of Nursing, Sun Yat-Sen University, NO. 74, Zhongshan 2nd Rd., Guangzhou, 510080 China

**Keywords:** Heart failure, Physical symptoms, Self-care

## Abstract

**Background:**

Patients with heart failure frequently report inadequate self-care behaviours. Physical symptoms can impact patients’ ability to perform self-care behaviours. However, studies investigating the association between physical symptoms and heart failure self-care behaviours have produced inconsistent findings, potentially due to variations in the determinants of self-care behaviours among patients with differing levels of self-care proficiency. Understanding the association between physical symptoms and self-care behaviours in heart failure patients with inadequate self-care behaviours could improve care for this subpopulation. The study aimed to explore the association between physical symptoms and self-care behaviours in Chinese heart failure patients with inadequate self-care behaviours.

**Methods:**

This analysis was based on primary data from a cross-sectional study that aimed to investigate factors associated with self-care in heart failure patients. Physical symptoms were measured using the Heart Failure Somatic Perception Scale. Self-care behaviours (i.e., self-care maintenance and management) of heart failure were measured using the Self-Care of Heart Failure Index (version 6.2). Patients who reported scores < 70 on both self-care maintenance and management behaviours were eligible and included in the analysis. Hierarchical regression analysis was performed to explore the association between physical symptoms and self-care behaviours.

**Results:**

A total of 189 patients were included in the analysis, with a mean age of 65 years and a median duration of living with heart failure of 24 months. Most participants were classified as New York Heart Association class III or IV. Dyspnoea symptoms were the most frequently reported physical symptoms. The results of the hierarchical regression analysis showed that the severity of physical symptoms was positively associated with self-care management behaviours (*β* = 0.157, 95% *CI*: 0.010, 0.368, *p* = 0.039) but not significantly associated with self-care maintenance behaviours (*β* = -0.133, 95% *CI*: -0.316, 0.026, *p* = 0.097).

**Conclusions:**

Based on the data collected in Changsha, China, we found that patients with HF with poor self-care experienced more dyspnoea symptoms. Severe HF physical symptoms might serve as drivers for better self-care management in patients with inadequate self-care behaviours. Effective care and support should be provided when physical symptoms worsen to facilitate patients’ engagement in self-care behaviour in this subpopulation.

**Supplementary Information:**

The online version contains supplementary material available at 10.1186/s12872-023-03247-2.

## Background

Heart failure (HF) has become a global pandemic, affecting approximately 26 million people worldwide and 4.5 million people in China alone [1, 2]. The primary goal of HF treatment is to achieve optimal management, which includes alleviating symptoms, improving prognosis, and reducing morbidity and mortality [3]. Self-care, defined as a naturalistic decision-making process that influences an individual's behaviours in maintaining physiologic stability (referring to self-care maintenance behaviours such as living a healthy lifestyle, adhering to the treatment regimen, and monitoring symptoms) and managing symptoms (referring to self-care management behaviours such as recognizing a change in health, implementing a treatment strategy, and evaluating the treatment implemented), has been regarded as the cornerstone of HF management [4, 5]. However, there are increasing concerns about inadequate self-care behaviours in people with HF. A study of 1,192 Italian adults with HF demonstrated that only 14.5% and 24.4% of the participants performed adequately in self-care maintenance and management behaviours, respectively [6]. In a Chinese study involving 321 patients with HF, only 7% of the participants showed adequate self-care maintenance behaviours, and 24% demonstrated adequate management behaviours [7]. Inadequate self-care behaviours have been linked to increased incidence of clinical events such as all-cause mortality, emergency room visits, and HF-related hospitalizations, as well as poorer quality of life [8, 9]. Considering the negative impact of inadequate self-care, it is crucial to identify and address the factors that affect the self-care behaviours of individuals who demonstrate inadequate self-care in both clinical practice and research endeavours.

People with HF experience a variety of symptoms, including physical, cognitive, and emotional symptoms, with physical symptoms being the most frequent, such as shortness of breath and swelling of feet, ankles, or legs [10, 11]. Physical symptoms are the main reason for costly hospitalizations, readmissions, emergency care, and death among patients with HF, imposing a substantial burden on patients, their families and health care systems [12, 13]. The importance of physical symptoms in disease management in patients with HF has been emphasized, especially in the context of China [4, 11, 14]. In Chinese patients with HF, physical symptoms are more prevalent than emotional symptoms, such as depression (e.g., approximately 90% and 44% of patients with HF have dyspnoea and depressive symptoms, respectively) [15]. Moreover, the stimuli from physical symptoms are significant drivers for Chinese patients to seek help or motivate them to take action to manage the disease [15].

Studies on the associations between physical symptoms and HF self-care behaviours are limited and yield inconsistent findings. Some studies reported that worse physical symptoms were associated with better HF self-care behaviours, suggesting that physical symptoms are drivers of self-care [16, 17]. In contrast, other studies reported that worse physical symptoms hindered the performance of self-care behaviours in patients with HF [18, 19]. Differences in patient characteristics, settings, and the measurements of self-care behaviours, as well as variations in the controlling factors employed among the above studies, may partially explain the mixed results. Researchers have also suggested that the determinants of self-care behaviours may differ based on patients’ level of self-care proficiency [20, 21]. Findings from previous studies exploring the relationship between physical symptoms and self-care behaviours conducted in HF patients without differentiating patients’ self-care proficiency level may not be generalized to patients with inadequate self-care behaviours [17, 20].

Therefore, this study primarily aimed to examine the association between physical symptoms and self-care behaviours in Chinese patients with HF who exhibit inadequate self-care behaviours. In addition, this study also examined other socio-demographic and clinical factors that may be associated with self-care behaviours in this subpopulation. Understanding the relationship between HF symptoms and self-care behaviours in the Chinese context would advance the care for patients with HF in Chinese culture and provide insights into planning effective self-care interventions for this particular population in real-life situations.

## Methods

### Study design

This analysis was based on primary data from a multicentre cross-sectional study. The aim of the primary study was to investigate the self-care behaviours of Chinese patients with HF and their associated psychological factors, such as self-care confidence, psychological resilience, hope, life orientation, and perceived stress [22]. This analysis was conducted to investigate the relationships between physical symptoms and self-care behaviours in patients with inadequate self-care behaviours who are underrepresented in previous studies to save time and resources.

### Sample and setting

In the primary study, 213 participants were recruited from cardiac wards in six public hospitals in Changsha, China, in 2017. They were eligible if they were: i) over 18 years old; ii) with a diagnosis of HF confirmed by physicians according to the Chinese guidelines for the Diagnosis and Treatment of Heart Failure 2014, which requires the presence of pulmonary and systemic congestion-related symptoms and signs such as dyspnoea, fatigue, or ankle swelling, a structural or functional cardiac abnormality as shown by ultrasound cardiogram, and elevated natriuretic peptide levels (BNP > 35 ng/L or NT-proBNP > 125 ng/L) [23]; iii) able to give consent; iv) able to complete the study, including speaking and reading Chinese. Patients with cognitive impairment or terminal illness (e.g., end-stage renal disease) indicated by their medical records were excluded. For this analysis, 189 out of 213 participants who had inadequate self-care behaviours indicated by self-care management and maintenance scores < 70 (measured by the Self-care of Heart Failure Index, SCHFI v.6.2) [24] were included.

This analysis used G*power 3.1.9.2 for sample size estimation using a linear multiple regression statistic test. The primary study showed 18.6% of the variance in self-care management explained by the factors of interest [22]. As only 12 predictors were included in this analysis, we conversely estimated an *R*^2^ of 0.10 for the expected equation model. Thus, a minimal sample size of 167 was needed (*α* = 0.05, power = 0.80).

### Measures and measurements

#### Sociodemographic and clinical characteristics

A self-developed questionnaire was used to collect the sociodemographic and clinical characteristics through medical record reviews and patient interviews. The sociodemographic characteristics collected included age, sex, educational level, marital status, and employment status. The clinical characteristics included months since being diagnosed with HF, hospital admission in the past year (yes/no), Charlson Comorbidity Index (CCI), and the New York Heart Association (NYHA) class.

#### Physical symptoms of heart failure

The Heart Failure Somatic Perception Scale (HFSPS v.3) [25] was used to measure participants’ physical symptoms. The HFSPS v.3 consists of 18 items measuring four types of physical symptoms, including dyspnoea (6 items), oedema (3 items), chest discomfort (2 items), and early and subtle symptoms (7 items). Patients responded on a Likert-6 scale (from 0 = *I did not have this symptom* to 5 = *extremely bothersome*) to indicate how much they were bothered by those symptoms in the past week. The total score ranges from 0 to 90, with a higher score indicating worse symptoms. The original HFSPS v.3 had an acceptable internal consistency with a Cronbach's *α* of 0.90 for the whole scale [25]. The Chinese version of HFSPS v.3 was provided by its original author, of which Cronbach's *α* was 0.85 in the sample of the primary study.

#### Self-care behaviours of heart failure

Self-care behaviours of heart failure were measured with the Self-care of Heart Failure Index (SCHFI v.6.2), a quantitative, ordinal, self-report, performance-rating scale [24]. The SCHFI v.6.2 scale consists of three subscales: the self-care maintenance subscale, the self-care management subscale, and the self-care confidence subscale, where self-care maintenance and self-care management constitute self-care behaviours [24]. The scale uses a Likert-4 scale, and each subscale score is standardised to a 0–100 range, with a higher score indicating better self-care behaviours [24].

The self-care maintenance subscale consists of 10 items measuring how often patients perform symptom monitoring and treatment adherence behaviours. The formula used to compute a standardised subscale score is (sum of items—10) × 3.333. A cut-off score of 70 or above was used to determine the adequacy of self-care maintenance behaviours. The self-care management subscale consists of 6 items measuring how patients recognize and respond to symptoms and evaluate the effectiveness of remedies. A standardised score is calculated using the formula: (sum of items—4) × 5 [24], with a cut-off score of 70 or above used to determine the adequacy of self-care management behaviours. The Chinese version of the SCHFI v.6.2 has been validated in the Chinese HF population, with acceptable internal consistency reported for the self-care maintenance and management subscales (Cronbach's *α* of 0.66 and 0.74, respectively) [26]. The construct validity was tested using confirmatory factor analysis, and the overall model fit was adequate both in the original English and the Chinese versions [24, 26].

### Data collection

A senior nurse in each sampled ward was appointed to screen electronic medical records to identify potential participants. The diagnoses of HF were also double-confirmed by the physicians. Then, the researcher assessed the eligibility of the potential participants identified by the nurses according to the inclusion and exclusion criteria. Eligible patients were invited to participate and informed of the purposes and procedures of the study. After obtaining written consent, participants were asked to complete the questionnaires independently. All data were collected by one researcher on the spot.

### Statistical analysis

Data analyses were performed using SPSS version 25. All continuous variables were examined for normal distribution. If they were normally distributed, means and standard deviations were used to describe continuous variables such as age, physical symptoms, and scores of self-care subscales (self-care maintenance and self-care management); otherwise, medians with interquartile ranges were used. We also described participants’ characteristics (e.g., sex and education background) and their responses to each item related to self-care behaviours with percentages. We performed hierarchical linear regressions to determine the associations between physical symptoms and self-care behaviours. In Model 1, the sociodemographic factors (i.e., age, sex, educational levels, marital status, and employment status) and clinical factors (i.e., months since being diagnosed with HF, hospital admissions in the past year, CCI, and NYHA classes) were entered. Then, in Model 2, we added physical symptoms based on Model 1. We did not introduce psychological factors such as self-care confidence or perceived stress as covariates in the regressions due to the potential mediation of these variables between physical symptoms and self-care behaviours, which violates the independence principle for covariates [27–29]. The *R*^*2*^ between Model 1 and Model 2 was compared to indicate the variance of change in self-care behaviours contributed by physical symptoms. The statistical significance level was set at *p* < 0.05 using a two-tailed test.

## Results

### Sociodemographic and clinical characteristics

The sociodemographic and clinical characteristics of the sample in this analysis are presented in Table 1. The mean age of the sampled participants was about 65 years old, and 37.6% of the samples were female. Nearly half of the participants held an education level of primary school or below (42.9%). The majority of the participants were currently married (83.6%) and unemployed (86.2%). Almost all the participants had been hospitalized due to HF in the past year (95.2%). Participants were diagnosed with HF for a median of 24 months and had an average CCI of 3.8. Most of the participants were in NYHA class III or IV (85.7%). The characteristics above were similar to the sample in the primary study (Supplementary Table S1).

**Table 1:** Sociodemographic and clinical characteristics, physical symptoms, and self-care behaviours among patients in the current analysis and the primary study (*N* = 189)

Characteristics	Mean ± SD/Median (IQR)	n (%)
Age	64.88 ± 12.77	
Sex (Female)		71 (37.6)
Education background		
Primary school and below		81 (42.9)
High school		91 (48.1)
College and above		17 (9.0)
Marital status		
Being married		158 (83.6)
Single/widowed/divorced		31 (16.4)
Employment status (Unemployed)		163 (86.2)
Months since HF diagnosis	24 (3–60)^a^	
Hospitalized due to HF in the past year (Yes)		180 (95.2)
CCI	3.83 ± 1.47	
NYHA class		
II		27 (14.3)
III		84 (44.4)
IV		78 (41.3)
Physical symptoms score (total range: 0–90)	25.89 ± 12.65	
Dyspnoea (range: 0–30)	11.61 ± 6.51	
Oedema (range: 0–15)	4.31 ± 2.79	
Chest discomfort (range: 0–10)	3.30 ± 2.27	
Subtle early symptoms (range: 0–35)	6.22 ± 3.77	

*Abbreviations*: *CCI* Charlson Comorbidity Index, *HF* Heart failure, *IQR* Interquartile range, *NYHA* New York Heart Association

^a^presented as the median (IQR)

### Physical symptoms of heart failure

The mean score of overall physical symptoms was 25.89 ± 12.65 (ranging from 0 to 90, with a higher score indicating worse symptoms). The highest-scored symptoms were related to dyspnoea (e.g., “I could not catch my breath”, “It was hard for me to breathe”, and “I could not do my usual activities because I was short of breath”). The lowest scored symptoms were related to oedema and subtle early symptoms (e.g., “I gained weight in the past week”, “My clothes felt tighter around my waist”, and “My shoes were tighter than usual at the end of the day”) (Table 1, Supplementary Table S2).

### Self-care behaviours of heart failure

**Fig. 1:**
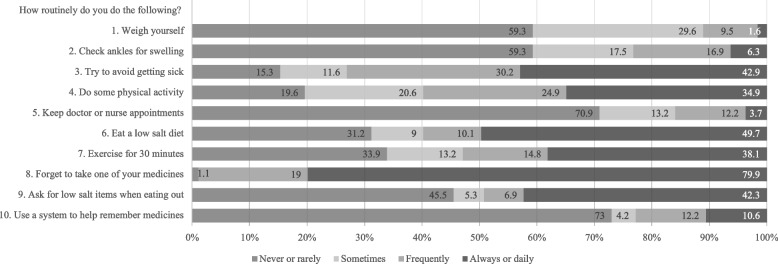
Participants’ self-care maintenance behaviours (*N* = 189)

Self-care maintenance. The average score for self-care maintenance was 45.59 ± 13.74, far below the cut-off point of 70 for adequate self-care behaviour. Most of the participants (76.8% to 98.9%) maintained poor behaviour regarding self-checking, taking medicines, and keeping doctor or nurse appointments. Approximately half of the participants did the physical activity (40.2%) or exercise (47.1%) and ate a low-salt diet (40.2%) or asked for low-salt items when eating out (50.8%) infrequently (Fig. 1).

**Fig. 2:**
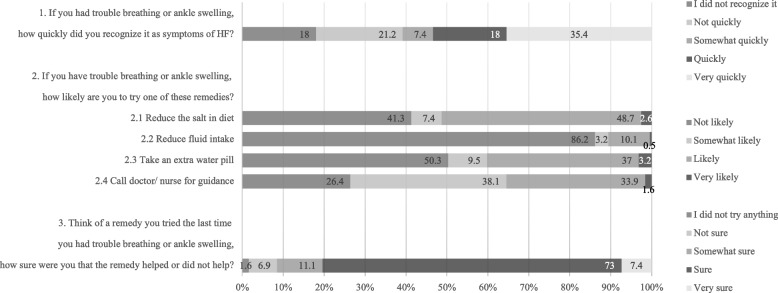
Participants’ self-care management behaviours (*N* = 189)

Self-care management. The average score for self-care management was 42.54 ± 15.18. Approximately half (53.4%) of the participants recognized the symptoms of HF quickly or very quickly. In response to the HF symptoms, approximately half of the participants were unlikely to reduce the salt in their diet (41.3%) or take an extra water pill (50.3%); even worse, most of them were unlikely to reduce fluid intake (86.2%). However, 80.4% of the participants reported that they were sure or very sure about the effectiveness of the remedies they tried last time in relieving their symptoms (Fig. 2).

### Heart failure physical symptoms and self-care behaviours for heart failure

The results of hierarchical regressions demonstrated that physical symptoms were not significantly associated with self-care maintenance (*β* = -0.133, 95% CI: -0.316, 0.026, *p* = 0.097; Table 2). Worse physical symptoms were associated with better self-care management (*β* = 0.157, 95% CI: 0.010, 0.368, *p* = 0.039; Table 3), which explained an additional 2.0% of the variance in self-care management.

**Table 2:** Predictors of self-care maintenance with hierarchical regression analysis (standardised coefficients *β* (95% confidence interval); *N* = 189)

Predictor variables	Model 1	Model 2
Age	0.084 (-0.111, 0.293)	0.093 (-0.100, 0.302)
Sex (0: male; 1: female)	-0.042 (-5.443, 3.071)	-0.037 (-5.289, 3.188)
Educational background_ high school (with reference to primary school)	0.158^*^ (0.043, 8.651)	0.150 (-0.168, 8.414)
Educational background_ college or above (with reference to primary school)	0.173^*^(0.794, 15.777)	0.163^*^ (0.340, 15.289)
Marital status (0: single/divorced/widow; 1: being married)	0.105 (-1.731, 9.576)	0.093 (-2.176, 9.124)
Employment (0: unemployed; 1: employed)	0.020 (-5.621, 7.198)	0.012 (-5.919, 6.859)
Months since HF diagnosis	0.153^*^ (0.001,0 .073)	0.170^*^ (0.004, 0.078)
HF associated hospitalization in the past year (0: no; 1: yes)	0.037 (-7.142, 11.861)	0.055 (-6.036, 13.070)
CCI	-0.062 (-2.202, 1.033)	-0.058 (-2.158,1.062)
NYHA_III (with reference to NYHA II)	-0.065 (-7.881, 4.285)	-0.039 (-7.186,5.040)
NYHA_IV (with reference to NYHA II)	-0.112 (-9.347, 3.122)	-0.064 (-8.180, 4.620)
Physical symptoms		-0.133 (-0.316, 0.026)
R^2^	0.083	0.098
R^2^ change	0.083	0.014

*Abbreviations*: *CCI* Charlson Comorbidity Index, *HF* Heart failure, *NYHA* New York Heart Association

^*^p < 0.05

^**^p < 0.01

**Table 3:** Predictors of self-care management with hierarchical regression analysis (standardised coefficients *β* (95% confidence interval); *N* = 189)

Predictor variables	Model 1	Model 2
Age	0.009 (-0.201, 0.223)	-0.001 (-0.212, 0.209)
Sex (0: male; 1: female)	-0.083 (-7.070, 1.888)	-0.088 (-7.208, 1.673)
Educational background_ high school (with reference to primary school)	-0.029 (-5.419, 3.638)	-0.020 (-5.093, 3.897)
Educational background_ college or above (with reference to primary school)	0.068 (-4.284, 11.481)	0.080 (-3.618, 12.044)
Marital status (0: single/divorced/widow; 1: being married)	0.143 (-0.020, 11.876)	0.157 (0.594, 12.432)^*^
Employment (0: unemployed; 1: employed)	-0.016 (-7.430, 6.058)	-0.006 (-6.964, 6.422)
Months since HF diagnosis	0.327 (0.048, 0.125)^**^	0.306 (0.043, 0.120)^**^
HF associated hospitalization in the past year (0: no; 1: yes)	0.040 (-7.123, 12.872)	0.019 (-8.643, 11.372)
CCI	0.113 (-0.524, 2.880)	0.109 (-0.556, 2.817)
NYHA_III (with reference to NYHA II)	0.041 (-5.145, 7.655)	0.010 (-6.095, 6.713)
NYHA_IV (with reference to NYHA II)	-0.158 (-1.687, 11.432)	0.102 (-3.571, 9.838)
Physical symptoms		0.157^*^ (0.010, 0.368)
R^2^	0.167	0.187
R^2^ change	0.167^**^	0.020^*^

*Abbreviations*: *CCI* Charlson Comorbidity Index, *HF* Heart failure, *NYHA* New York Heart Association

^*^p < 0.05

^**^p < 0.01

Furthermore, we found that compared to individuals with a primary education level, those with a college education or higher had better self-care maintenance (Table 2). Additionally, the length of living with HF was positively associated with both self-care maintenance and management (Tables 2 and 3).

## Discussion

The findings of this analysis revealed a statistically significant association between worse physical symptoms and better self-care management in HF patients living in Changsha, China with inadequate self-care behaviours, although the effect size was small. This suggested that symptom severity could serve as an essential driver of self-care management in patients with HF who have inadequate self-care behaviours. Further studies are needed to identify more predictors of self-care behaviours in this subpopulation.

### Self-care behaviours in Chinese patients with HF

The average self-care maintenance and management scores of participants in this analysis were lower than those reported in previous studies conducted in mainland China [7], Hong Kong [30], and Italy [6]. This is understandable since our analysis only focused on participants with inadequate self-care behaviours. This analysis revealed that more than half of the participants could quickly recognize their symptoms (e.g., shortness of breathing or ankle swelling) as being related to HF, but they did not reduce their fluid intake or salt consumption in their diets, nor did they take an extra diuretic in response to these symptoms. This suggests that correct symptom recognition does not necessarily translate into the initiation of proper actions to manage those symptoms. Moreover, the participants’ inadequate self-care behaviours may be attributed to their low education levels, which can lead to inadequate knowledge and skills related to self-care. This is consistent with another Chinese study which reported that HF patients with low education levels lacked knowledge about proper diet and had difficulty understanding healthcare providers’ instructions, resulting in poor performance of self-care behaviours [31].

Cultural beliefs and social norms may also influence the self-care behaviours of patients with HF, as indicated by other studies [31, 32]. Many Chinese people tend to hold the beliefs of “let it be” and “human beings are born to suffer” towards life and health. People, therefore, usually have little incentive to perform self-care or change their harmful habits when suffering from health problems [31, 32]. Additionally, it is common for Chinese people to live with extended families, where requests such as asking for an additional low-salt meal may be regarded as causing trouble or inconvenience to other family members [31]. Thus, this may lead to poor self-care maintenance behaviour in sodium restriction. Other contextual factors, such as the accessibility of other remedies (e.g., traditional Chinese medicine) and the availability of resources for HF management, social support, healthcare system, would also influence people's decision-making in self-care behaviours [33–35]. Therefore, contextual factors should be considered when planning effective interventions to improve patients’ self-care behaviours.

### Physical symptoms in Chinese patients with HF

Few studies have investigated physical symptoms in Chinese patients with HF. Our study reported an overall score of 28.6 on HFSPS (v.3), which was higher than that of outpatients with HF (mean = 24.3) [13] but lower than that of patients hospitalized with recurrent symptoms (mean = 37.5) [36]. Patients with HF in different settings had varied compositions of symptom profiles. The current analysis showed that symptoms related to dyspnoea were the most commonly reported, whereas subtle early symptoms were the least frequently reported by the hospitalized and symptomatic participants, which was similar to the findings of Altice and Madigan [36]. However, Jurgens and colleagues investigated community-dwelling patients who had stable HF conditions and reported opposite findings: patients with HF experienced more early and subtle symptoms but fewer symptoms associated with dyspnoea [25]. The possible explanation might be the difference in heart condition between the two samples since the participants in our analysis were hospitalized patients who were more symptomatic and generally had more deteriorated cardiac conditions. In contrast, Jurgens and colleagues studied community-dwelling patients with HF who generally had stable and less severe heart conditions.

### Association between physical symptoms and self-care behaviours

Our study found that in Chinese HF patients living in Changsha with inadequate self-care behaviours, worse HF physical symptoms were associated with better self-care management behaviours. This is consistent with the findings of some previous studies where participants’ self-care management was suboptimal [16, 17]. Studies have shown that patients with HF with poor self-care behaviours are motivated to initiate self-care management only when they experience worsened symptoms [20, 37], indicating that symptom severity is an essential driver of self-care management. Unlike our study, some studies did not report any associations between HF physical symptoms and self-care management in a sample of community HF patients without differentiating their self-care behaviour proficiency [25, 36]. The inconsistency might be because community patients included in these studies generally experienced more subtle symptoms compared to the patients in our analysis, which might not be recognized as signs of an exacerbation of HF and, thus, failed to drive action to manage these symptoms [25]. Future studies are warranted to explore the specific symptoms that motivate patients to initiate self-care management behaviours.

Some previous studies reported that HF patients were more engaged in self-care maintenance behaviours when they were more bothered by their symptoms [10, 16]. However, unlike these studies, our analysis did not find significant associations between physical symptoms and self-care maintenance behaviours. One possible explanation for this could be self-care maintenance behaviours are long-established actions that individuals take to maintain physiologic stability [4, 5], and physical symptoms alone may not account for much variance in the self-care maintenance level in this sample of patients, who generally had poor self-care maintenance behaviours. There might be other factors influencing self-care maintenance behaviours among Chinese patients, especially those with inadequate self-care maintenance behaviours, which should be further explored.

Several limitations should be considered when interpreting the results of this study. First, although many predictors of self-care behaviours of HF were included in the models, the degree of variance explained by these predictors was generally low (from 11.8% to 18.4%). Psychological predictors such as depressive symptoms, which may also affect patients' decision-making on self-care, were not included in this analysis considering their potential mediation effects between physical symptoms and self-care behaviours and the lack of data in the primary study. Other factors that contribute most to self-care behaviours in HF patients should be explored in future studies. Second, the nature of the cross-sectional study design limits the inference of any causal relationship between physical symptoms and self-care behaviours. Third, this study only included patients who performed poorly in self-care behaviours in a single city of China, namely Changsha, and primarily included participants with NYHA functional classes II to IV. The generalization of the findings to patients with HF with good self-care proficiency, and better cardiac function, from other regions of China or other countries, should be made with caution. Fourth, due to the lack of data on cognitive function in the primary study, we were unclear if cognitive function interferes with the association between physical symptoms and self-care behaviours. Further studies are expected to explore the role of cognitive function in HF self-care behaviours. Additionally, Cronbach's *α* for the SCHFI v.6.2 is lower than the desired reliability estimates for an established instrument. Future studies are thus warranted to examine the psychometric properties of the SCHFI v.6.2 or adopt other instruments with sounder validity and reliability to measure self-care behaviours.

### Implications for further practice and recommendations for further research

Our study has important implications for practice and research. For clinicians and nurses, it is important to differentiate patients with different levels of HF self-care proficiency and identify those who do not manage their disease well to provide effective care. In Chinese HF patients with inadequate self-care behaviours, timely and appropriate assistance, support, and guidance should be provided when patients become physically symptomatic, which might be when patients are more willing to take an active role in self-care behaviours. In addition, healthcare instructors should teach patients the knowledge and skills to recognize subtle physical symptoms and adopt motivational techniques to promote patients' engagement in self-care behaviours.

Regarding the recommendations for further research, future studies with rigorous designs, large sample sizes, including patients from more regions and countries, and adopting other instruments with sounder validity and reliability to measure self-care behaviours are needed to confirm our findings and explore more predictors, such as psychological or cognitive factors, influencing self-care behaviours. More importantly, the mechanism underlying the relationship between physical symptoms and self-care management in patients with HF with poor self-care behaviours could be further elaborated.

## Conclusions

With the data collected in Changsha, China, we found that patients with HF with poor self-care experienced more dyspnoea symptoms. Severe HF physical symptoms might serve as drivers for better self-care management in patients with inadequate self-care behaviours. Thus, effective care and support should be provided when physical symptoms worsen to facilitate patients’ engagement in self-care behaviour in this subpopulation.

## Supplementary Information

**Additional file 1: Table S1.** Participants’ socio-demographic and clinical characteristics, physical symptoms, and self-care behaviours in the parental study. **Table S2.** Item score of physical symptoms measured by the Heart Failure Somatic Perception Scale.

## Data Availability

The dataset of the primary study is available at 10.7910/DVN/WENHL4.

## Abbreviations

CCI: Charlson comorbidity index
HF: Heart failure
HFSPS: Heart failure somatic perception scale
NYHA: New York Heart Association
SCHFI: Self-care of heart failure index
