# Supplementary material for: The association between physical symptoms and self-care behaviours in heart failure patients with inadequate self-care behaviours: a cross-sectional study
Source: BMC Cardiovasc Disord. 2023 Apr 22;23:205. doi: 10.1186/s12872-023-03247-2 (PMC10122378; doi:10.1186/s12872-023-03247-2)
Supplement: Supplementary file 1 — Additional file 1: Table S1. Participants’ socio-demographic and clinical characteristics, physical symptoms, and self-care behaviours in the parental study. Table S2. Item score of physical symptoms measured by the Heart Failure Somatic Perception Scale. [file 12872_2023_3247_MOESM1_ESM.docx]

| **Characteristics** | **Mean ± SD/ Median (IQR)** | **n (%)** |
| --- | --- | --- |
| Age | 65.42 ± 12.78 |  |
| Sex (Female) |  | 81 (38) |
| Education background |  |  |
| Primary school and below |  | 89 (41.8) |
| High school |  | 105 (49.3) |
| College and above |  | 19 (8.9) |
| Marital status |  |  |
| Being married |  | 176 (82.6) |
| Single/widow/divorced |  | 37 (17.4) |
| Employment status (Unemployed) |  | 187 (87.8) |
| Months since HF diagnosis | 24 (4-60)^a^ |  |
| Hospitalized due to HF in the past year (Yes) |  | 204 (95.8) |
| CCI | 3.89 ± 1.48 |  |
| NYHA class |  |  |
| II |  | 30 (14.1) |
| III |  | 95 (44.6) |
| IV |  | 88 (41.3) |
| Physical symptoms score (total range: 0-90) | 26.21 ± 12.98 |  |
| Dyspnea (range: 0-30) | 11.69 ± 6.63 |  |
| Edema (range: 0-15) | 4.35± 2.84 |  |
| Chest discomfort (range: 0-10) | 3.33 ± 2.29 |  |
| Early subtle (range: 0-35) | 6.33 ± 3.92 |  |
| Self-care maintenance (range: 0-100) | 47.88 ± 15.23 |  |
| Self-care management (range: 0-100) | 45.66 ± 17.28 |  |

**Supplementary Tables**

Table S1 Participants’ socio-demographic and clinical characteristics, physical symptoms, and self-care behaviours in the parental study (N=213)

Abbreviations: CCI: Charlson Comorbidity Index; HF: Heart failure; IQR: Interquartile ranges; NYHA: New York Heart Association. Note: ^a^ presented as median (IQR).

Table S2 Item score of physical symptoms measured by the Heart Failure Somatic Perception Scale (HFSPS v.3)

| **Item** | **Score**  **(Mean ± SD)** | **Rank** |
| --- | --- | --- |
| 1. I could feel my heartbeat get faster | 1.49±1.54 | 7 |
| 2. I could not breathe if I lay down flat | 1.42± 1.59 | 8 |
| 3. I felt discomfort or pain in my chest | 1.85± 1.45 | 5 |
| 4. I had an upset stomach | 0.87±1.24 | 15 |
| 5. I had a cough | 1.27±1.30 | 12 |
| 6. I was tired | 2.09±1.36 | 4 |
| 7. I could not catch my breath | 2.70±1.30 | 1 |
| 8. My feet were swollen at the end of the day | 1.37±1.40 | 10 |
| 9. I woke up at night because I could not breathe | 1.28±1.52 | 11 |
| 10. My shoes were tighter than usual | 0.58±1.07 | 16 |
| 11. I gained weight in the past week | 0.15±0.59 | 18 |
| 12. I could not do my usual activities because of SOB | 2.40±1.67 | 3 |
| 13. Getting dressed made it hard to breathe | 1.22± 1.47 | 14 |
| 14. My clothes felt tighter around my waist | 0.50±1.08 | 17 |
| 15. I woke up at night because I had to urinate | 1.41±1.21 | 9 |
| 16. I had to rest more than usual during the day | 1.68±1.37 | 6 |
| 17. It was hard for me to breathe | 2.68±1.36 | 2 |
| 18. I did not feel like eating | 1.25±1.41 | 13 |
